# An endothelial rejection line following Descemet stripping automated endothelial keratoplasty

**DOI:** 10.1186/s12886-020-01575-x

**Published:** 2020-07-29

**Authors:** Muhannad Alkhalifah, Bader Alqahtani, Abdulmohsen Almulhim, Waleed Alsarhani

**Affiliations:** 1 Department of Ophthalmology, College of Medicine, King Saud University, Riyadh, Saudi Arabia; 2 Department of Ophthalmology, King Abdulaziz Medical City, Jeddah, Saudi Arabia; 3 Department of Ophthalmology, College of Medicine, Jouf University, Sakakah, Al-Jouf Saudi Arabia

**Keywords:** Corneal transplant, Graft rejection, DSAEK, Endothelial rejection line

## Abstract

**Background:**

The endothelial rejection line is rarely seen after Descemet stripping automated endothelial keratoplasties (DSAEKs). Here, we present a case of endothelial graft rejection with an endothelial rejection line occurring 1 year after the procedure.

**Case presentation:**

A 58-year-old female presented with graft rejection 1 year following a DSAEK procedure. The episode started when she tapered down her loteprednol to once a day. Slit-lamp examination showed a mildly injected conjunctiva with 1+ corneal oedema. On the posterior surface of the cornea, there was an endothelial rejection line (Khodadoust line) with keratic precipitates and multiple areas of anterior synechia.

**Conclusion:**

The classic endothelial rejection line should be kept in mind as a rare sign of DSAEK graft rejection.

## Background

The rate of graft rejection following endothelial keratoplasties is significantly lower than that following penetrating keratoplasties (PKPs) [1]. Hence, endothelial keratoplasties have largely replaced PKP in treating endothelial diseases such as Fuchs endothelial dystrophy and pseudophakic bullous keratopathy. Moreover, graft rejection after endothelial keratoplasty is entirely endothelial. Early recognition and treatment of endothelial graft rejection is of extreme importance. Signs of endothelial graft rejection include keratic precipitates (KPs), corneal oedema and an anterior chamber reaction. According to different studies on Descemet stripping automated endothelial keratoplasty (DSAEK) complications, the 3-year incidence of immunologic graft rejection episodes ranges from 4 to 22% [1–5]. A recent study by Price et al. found that endothelial rejection occurs in 7.9% of DSAEK cases over a 5-year period [6]. A number of studies have reported no endothelial rejection lines [2–4], although two studies have reported these lines [1, 7]. Here, we present a case of endothelial graft rejection with an endothelial rejection line occurring 1 year after DSAEK.

## Case presentation

**Fig. 1:**
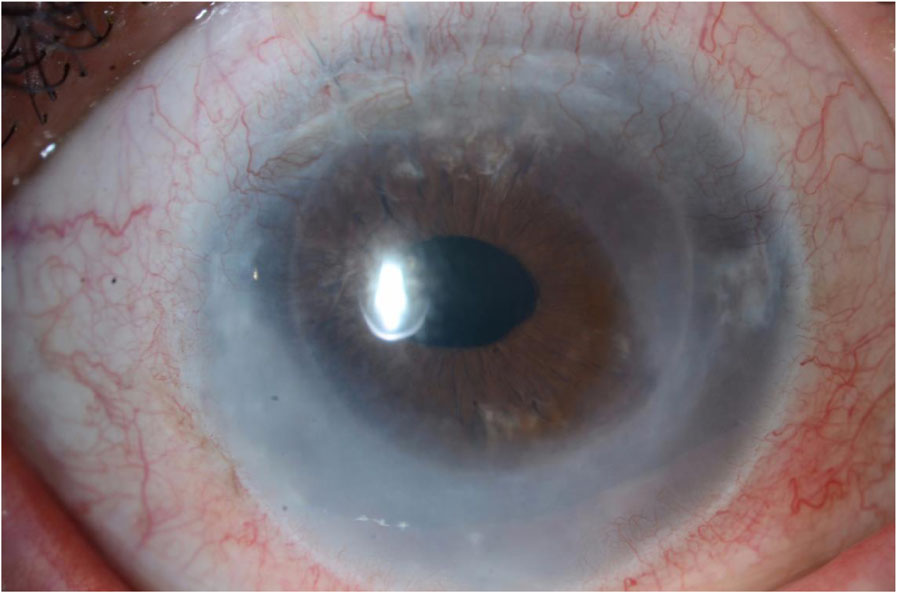
Diffuse corneal oedema in a grafted eye

**Fig. 2:**
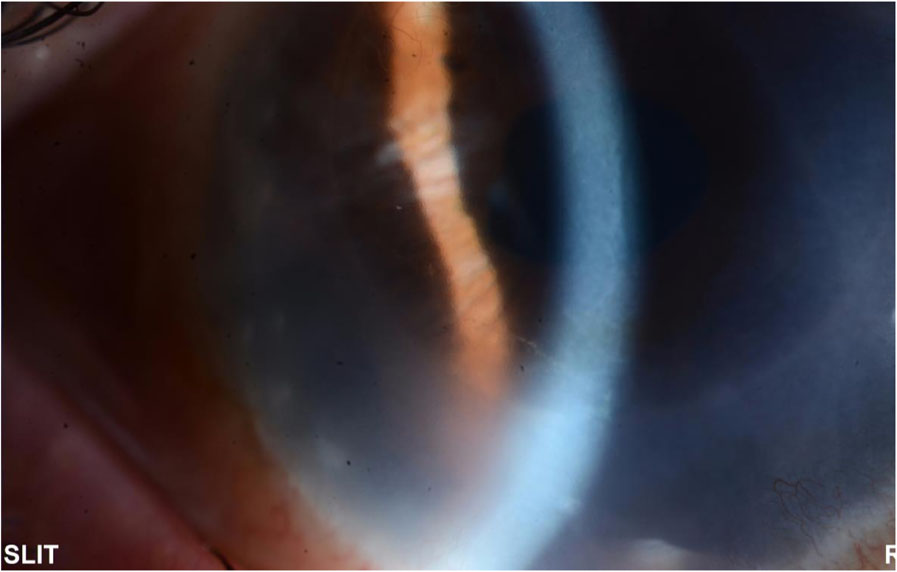
Endothelial rejection line (Khodadoust line)

**Fig. 3:**
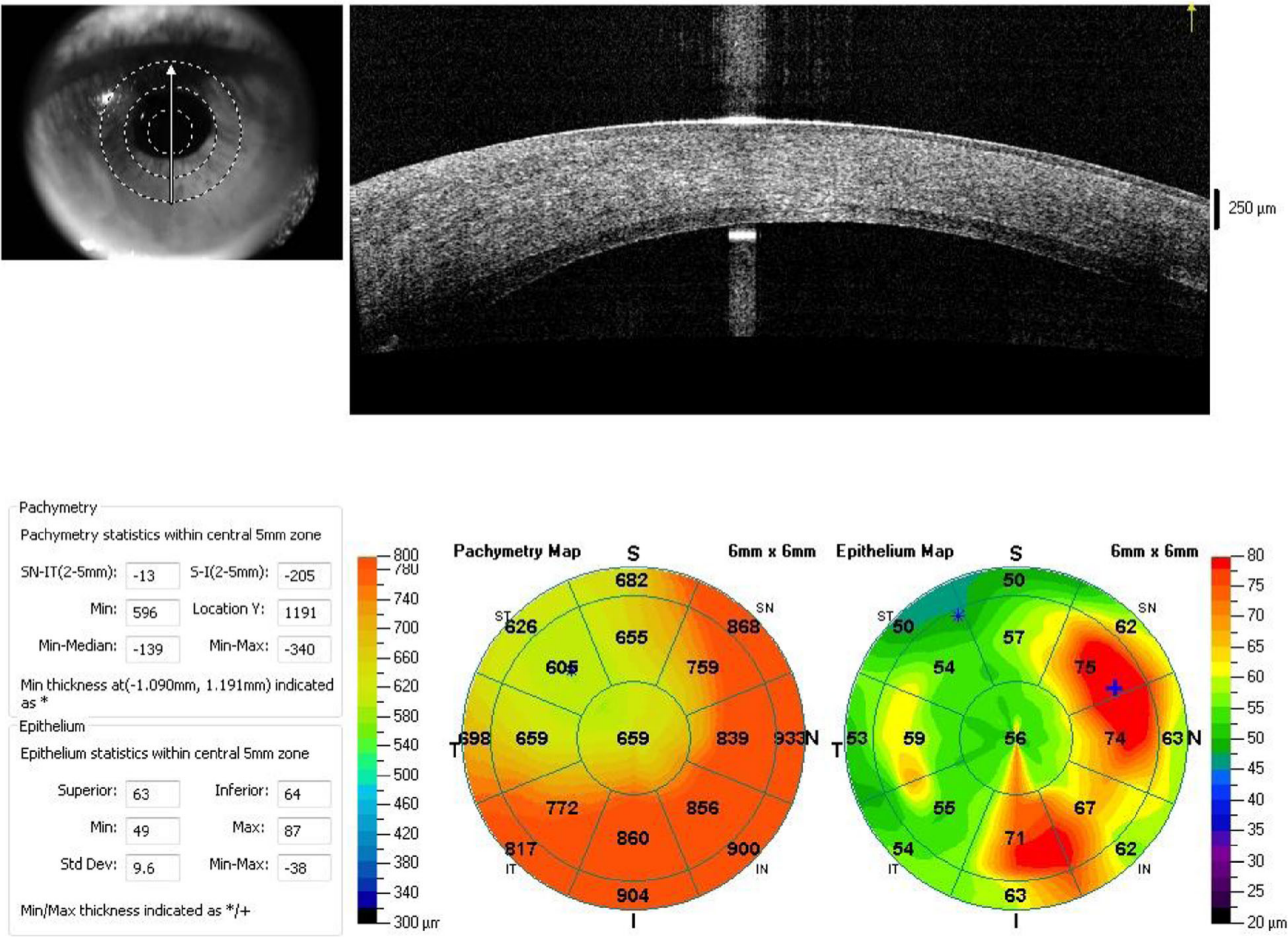
Anterior segment optical coherence tomography showing an attached lenticule with a central corneal thickness of 659 μm

**Fig. 4:**
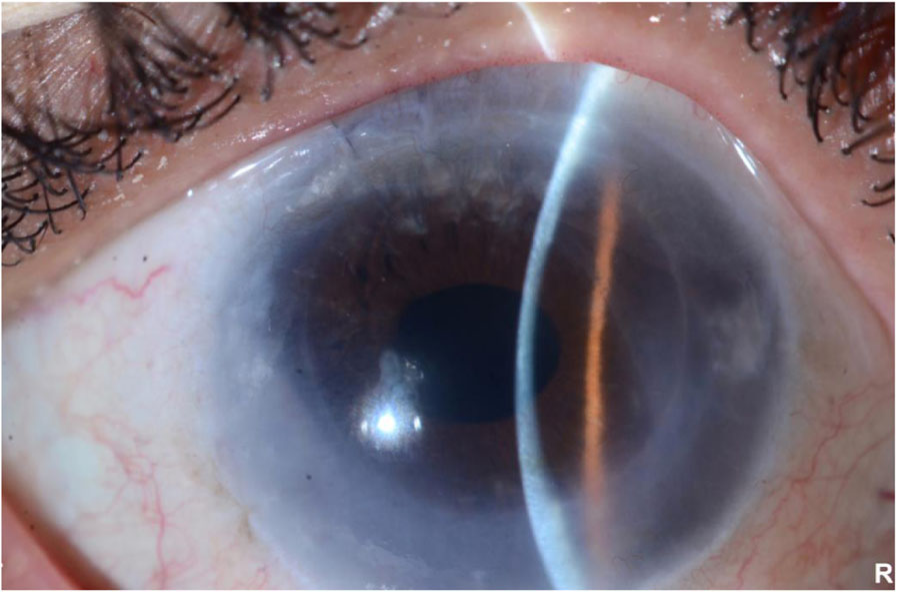
Improvement in corneal oedema 4 weeks after diagnosis and treatment

A 58-year-old female presented with a one-week history of blurred vision associated with photophobia and redness. The episode started when she tapered her loteprednol from twice a day to once a day. The patient underwent DSAEK regrafting 1 year before her presentation. Her first DSAEK procedure had been performed 4 years prior for a decompensated cornea secondary to an iris-fixated anterior chamber lens. Her best corrected visual acuity in the right eye was 20/200, and the intraocular pressure was 9 mmHg. Slit-lamp examination showed a mildly injected conjunctiva with 1+ corneal oedema (Fig. 1). On the posterior surface of the cornea, there was an endothelial rejection line (Khodadoust line) with KPs extending from 4 to 8 o’clock (Fig. 2). Additionally, there were multiple areas of anterior synechia. The pupil was irregular and oval in shape, and the anterior chamber was deep with occasional cells. Examination of the left eye was unremarkable. The patient had a central corneal thickness of 659 μm (measured by anterior segment optical coherence tomography) on initial presentation (Fig. 3). The diagnosis of graft rejection was made, and the patient was started on prednisolone acetate 1% drops every 1 h. After 1 month of follow-up, the patient’s vision improved from 20/200 to 20/60, and the corneal oedema also improved (Fig. 4).

## Discussion and conclusion

The rate of graft rejection after DSAEK is significantly lower than that after PKP [4, 8]. This is explained by the lower number of sutures, the absence of graft exposure to ocular-surface antigen-presenting cells, the lack of contact with stromal blood vessels, and the fact that DSAEK involves less immunogenic donor tissue than PKP [8, 9]. In a study performed by Basak et al., two-thirds of patients who experienced graft rejection in a period between 1 and 3 years after surgery complained of blurred vision and photophobia, while the other one-third presented graft rejection that was discovered incidentally during a routine follow-up clinical evaluation. All of the patients who had DSAEK rejection exhibited KPs, anterior chamber cells, and diffuse corneal oedema. However, none of these cases developed an endothelial rejection line [2].

The endothelial rejection line (Khodadoust line) is an aggregation of lymphoid cells on the corneal endothelial side and is a sign of immunologic corneal allogenic graft rejection [10], which has been classically described after PKP. In a study by Jordan et al., no cases of an endothelial rejection line were reported among their 54 patients who experienced immunological graft rejection episodes after DSAEK [3]. The researchers noted that stromal vascularization is often associated with the site of an endothelial rejection line in PKP, so the comparative absence of stromal vessels extending into DSAEK tissue may reduce the likelihood of an endothelial rejection line. In our patient, iridocorneal adhesions possibly triggered immune rejection of the graft. Three to four quadrants of anterior synechia have been reported to be a strong risk factor for graft rejection [11]. In murine models, anterior synechia triggered a cytotoxic T lymphocyte response with more cytokine expression and eventually a higher rejection rate compared to models without anterior synechia [12]. Saelens et al. and Fiorentzis et al. reported two cases of endothelial rejection line following DSAEK with no stromal vascularization, similar to our case, but with no anterior synechia [7, 13]. Therefore, we believe stromal vascularization and anterior synechia are factors that may increase the likelihood of the formation of the rejection line, but they do not have to be present.

In our patient, graft rejection with the endothelial rejection line was encountered 1 year post-DSAEK shortly after she tapered the corticosteroid drops from twice daily to once daily. Our patient was treated with an aggressive topical steroid regimen, and her visual acuity improved over a short period of time. This shows the importance of early recognition and prompt treatment for a favourable clinical outcome. It is of vital importance to instruct the patient undergoing DSAEK regarding the careful use of postoperative medications and the consequences of poor compliance. In our patient, the rejection episode could be attributed to tapering the topical steroids. In the case of an endothelial rejection line following DSAEK that was reported by Saelens et al., the patient had stopped the steroid drops 1 month after her operation [7]. The cessation of steroids postoperatively was found to be the most predictive factor for the development of DSAEK rejection, with a 5.5-fold greater risk, as shown by Wu et al. [1]. A similar observation was reported by Sepsakos et al. [4], as termination of steroid use was found to be the strongest risk factor for graft rejection. However, a more recent study did not find topical corticosteroid termination to be a significant factor predisposing to endothelial rejection after DSAEK [5]. In addition, close follow-up, especially in the first postoperative year, and subsequent long-term follow-up have been found by Wu et al. to be the most important factor in varying immunologic graft rejection rates [1].

As we described, the present case shows that the endothelial rejection line is a rare but important sign of endothelial rejection following DSAEK. Furthermore, the present case raises the possibility that anterior synechia may trigger the formation of Khodadoust lines. Since this is a single case report, it is difficult to explain the underlying mechanism and risk factors associated with the endothelial rejection line following endothelial keratoplasties. Indeed, the classic endothelial rejection line should be kept in mind as a rare sign of DSAEK graft rejection.

## Data Availability

Data sharing was not applicable to this article, as no datasets were generated or analysed during the current study.

## Abbreviations

*PKP*: Penetrating keratoplasty
*DSAEK*: Descemet stripping automated endothelial keratoplasty
*KP*: Keratic precipitate
